# The HD-Domain Metalloprotein Superfamily: An Apparent Common Protein Scaffold with Diverse Chemistries

**DOI:** 10.3390/catal10101191

**Published:** 2020-10-15

**Authors:** Michelle Langton, Sining Sun, Chie Ueda, Max Markey, Jiahua Chen, Isaac Paddy, Paul Jiang, Natalie Chin, Amy Milne, Maria-Eirini Pandelia

**Affiliations:** Department of Biochemistry, Brandeis University, 415 South Street, Waltham, MA 02453, USA

**Keywords:** HD-domain, metalloprotein, oxygenase, phosphatase, phosphodiesterase, hydratase, nucleic acid, phosphonate, diiron

## Abstract

The histidine–aspartate (HD)-domain protein superfamily contains metalloproteins that share common structural features but catalyze vastly different reactions ranging from oxygenation to hydrolysis. This chemical diversion is afforded by (i) their ability to coordinate most biologically relevant transition metals in mono-, di-, and trinuclear configurations, (ii) sequence insertions or the addition of supernumerary ligands to their active sites, (iii) auxiliary substrate specificity residues vicinal to the catalytic site, (iv) additional protein domains that allosterically regulate their activities or have catalytic and sensory roles, and (v) their ability to work with protein partners. More than 500 structures of HD-domain proteins are available to date that lay out unique structural features which may be indicative of function. In this respect, we describe the three known classes of HD-domain proteins (hydrolases, oxygenases, and lyases) and identify their apparent traits with the aim to portray differences in the molecular details responsible for their functional divergence and reconcile existing notions that will help assign functions to yet-to-be characterized proteins. The present review collects data that exemplify how nature tinkers with the HD-domain scaffold to afford different chemistries and provides insight into the factors that can selectively modulate catalysis.

## 1. Introduction

The histidine–aspartate (HD)-domain superfamily [1] (IPR003607) contains more than 318,000 metalloproteins that are involved in a wide array of functions including immunoresponse [2], nucleic acid metabolism [3–5], inflammation [6], virulence [6–8], stress response [9,10], and small molecule activation [11–13]. They are found across all domains of life and are typified by a tandem histidine–aspartate (HD) dyad that coordinates at least one (often two or three) metal ions. Although there are many uncharacterized HD-domain proteins, chemical diversion appears to be linked to details in the local protein environment, extra ligands, and genomic co-occurrence with partner proteins, all of which may serve as blueprints for their functional assignment.

### 1.1. Characteristics and General Classification of HD-Domain Proteins

**Figure 1.**
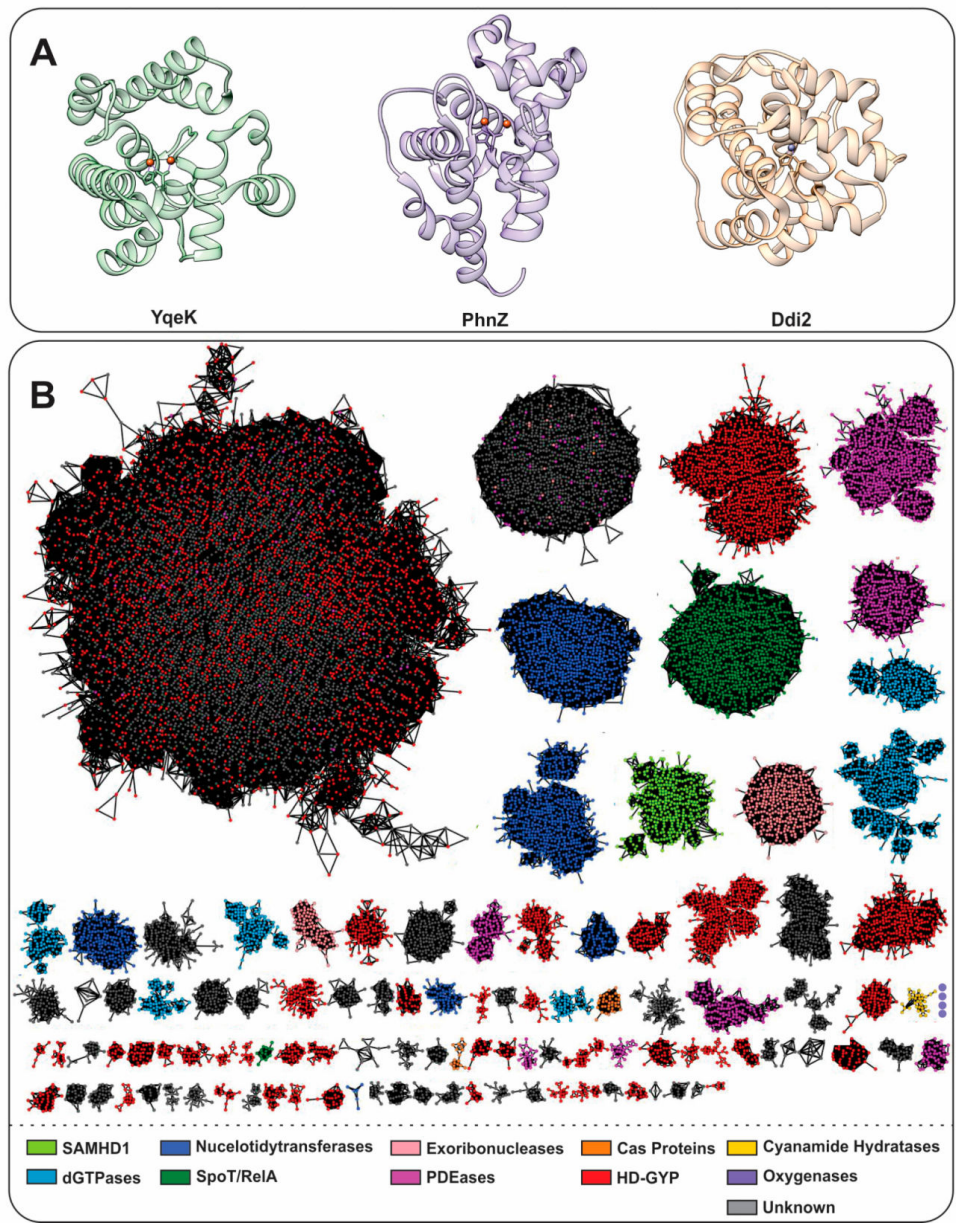
(**A**) Helical structure of three HD-domain proteins. YqeK (PDB: 2O08) is a phosphatase, PhnZ (PDB: 4N6W) is an oxygenase, and Ddi2 (PDB: 6DKA) is a lyase. All exhibit a helical fold characteristic to HD-domain proteins despite their diverse functions. (**B**) Sequence similarity network (SSN) of the HD-domain superfamily depicting its size and functional clustering. The SSN was generated via the Enzyme Function Initiatives-Enzyme Similarity tool (EFI-EST) and visualized in Cytoscape. The SSN was generated by employing the IPR003607 family and tailored so that nodes represent sequences with ≥ 50% identity and an e-value of 5. The SSN was further refined to contain the major protein clusters (size-wise), which amount to 183,015 unique protein sequences. Edges between nodes represent an alignment score of 70. HD-domain phosphohydrolases (SpoT/RelA, SAMHD1, deoxyguanosine phosphatases (dGTPases), nucleotidyltransferases) are represented in green and blue, while hydratases are shown in yellow. PDEs are shown in red (HD-GYP proteins), light pink (exoribonucleases), orange (Cas proteins), and pink (PDEases). Oxygenases are shown in purple and their cluster, which consists of four nodes (372 sequences), is enlarged for visualization. Gray clusters contain proteins of unidentified function.

The main traits that functionally differentiate HD-domain proteins are (i) the chemical nature of the metal ion/cofactor, (ii) cofactor nuclearity, (iii) supernumerary ligands, (iv) conserved amino acid sequence motif insertions or residues vicinal to the active site, (v) auxiliary domains with catalytic or regulatory roles, and (vi) interaction with protein partners. These six features appear to distinctly influence the range of chemistries these proteins perform. Despite variations in primary sequence, all HD-domain proteins share the HD residue dyad that coordinates transition metal ions such as Fe, Mn, Co, Mg, Cu, Zn, and Ni (Table 1) as well as a helical fold (Figure 1A) and functionally cluster based on sequence similarities (Figure 1B) [1,3,14].

**Figure 2.**
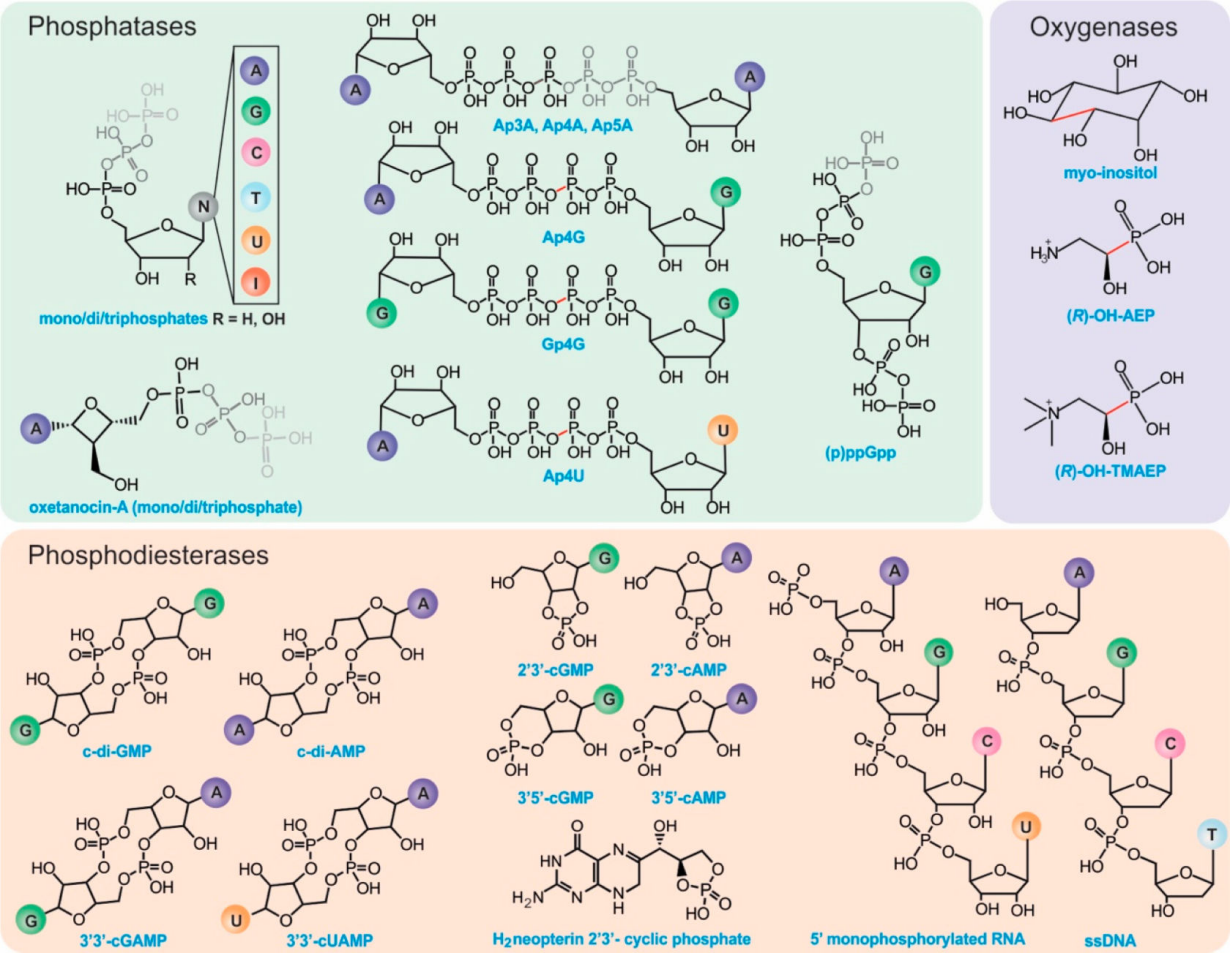
Known substrates of HD-domain proteins. Phosphatases can remove one to three terminal phosphate groups from (deoxy)ribonucleotides or cleave (a)symmetrically polyphosphate containing nucleotides (represented in gray). The position of cleavage has been highlighted in red for substrates with four phosphates. PDEs hydrolyze phosphodiester bonds of cyclic (di)nucleotide substrates via either one-step hydrolysis (cleavage of one side of the diester bond) releasing a linearized product or two-step hydrolysis releasing individual nucleotides. PDEs can also act on RNA and DNA substrates. HD-domain oxygenases cleave a C-X bond (indicated in red).

The HD-domain superfamily fosters three different enzymatic classes: hydrolases, oxygenases, and lyases, with the hydrolases being the largest and best-characterized group. Hydrolases are further subdivided into (i) phosphatases, including dGTPases [38], RelA/SpoT [9,17], SAMHD1 [18,19], EF1143 [20,21], etc. and (ii) phosphodiesterases (PDEs), including exoribonucleases [39], PDEases [27–34], Cas proteins [23–26], and HD-GYPs [14,35,36,40] (Figure 1B). Phosphatases hydrolyze a multitude of (deoxy)nucleotide-based substrates that vary in the identity of their base(s) and the extent of phosphorylation (Figure 2) [3,5,16,22]. PDEs, on the other hand, degrade a variety of cyclic signaling molecules and single-stranded nucleic acids (Figure 2) [23–25,41]. Oxygenases and lyases are relatively recent additions to the HD-domain superfamily with only a handful of representatives to have been biochemically characterized. All identified oxygenases catalyze the oxidative cleavage of C-C/P bonds [11–13,15] (Figure 2), while the single known lyase is a cyanamide hydratase [37]. This functional plurality widens the chemical repertoire of the HD-domain superfamily, which is likely to harbor more oxygenases, lyases, or enzymes with novel chemistries.

### 1.2. HD-Domain Hydrolases: The Phosphatase Subfamily

**Figure 3.**
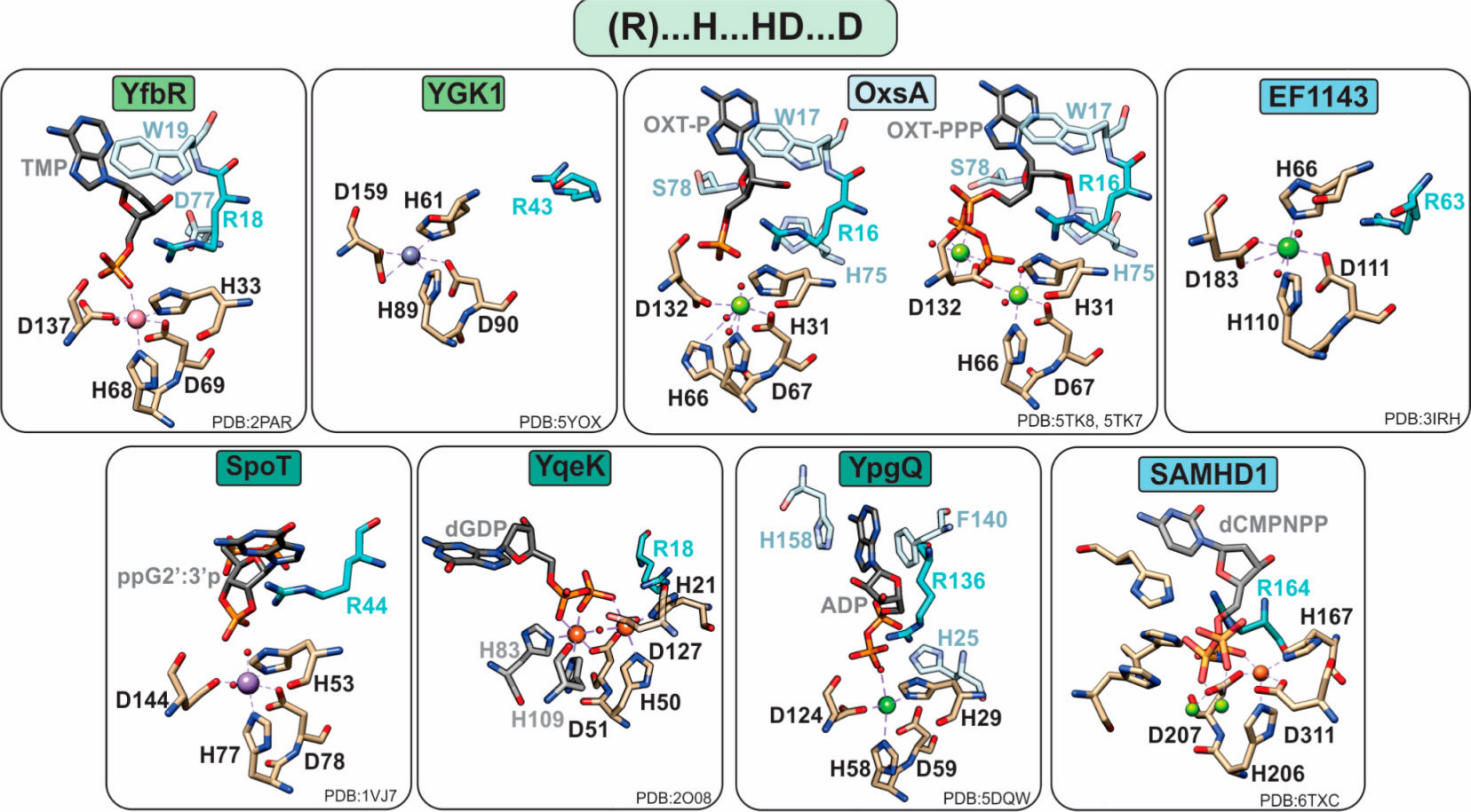
Active sites of HD-domain phosphohydrolases. Mononuclear HD-domain phosphohydrolases utilize a conserved motif “H…HD…D” to bind a variety of metals including cobalt (pink), zinc (purple), magnesium (light green), nickel (dark green), or iron (orange). Small red spheres represent water molecules. The dinuclear phosphatase YqeK harbors two extra histidines between the HD and D residues to stabilize the second metal ion. Phosphatases are classified into mono-, di-, or triphosphohydrolases, labeled in light green, dark green, and blue, respectively. All phosphohydrolases have a conserved arginine (shown in teal), which is located typically three residues prior to the first histidine of the HD motif and in the vicinity of the oxygens of the substrate phosphate group. Other important residues are shown in pale blue and are described in the text.

HD-domain phosphatases play essential roles in regulating the cellular pool of (deoxy)ribonucleotides and signaling molecules involved in bacterial stress responses [3,4,10,16,42], such as (p)ppGpp and Ap_4_A (Figure 2). Phosphatases are further subdivided into mono-, di-, and triphosphatases with distinct structural features that may provide clues for the future classification of unknown HD-domain phosphatases. Interestingly, all have a strictly conserved arginine residue prior to the first histidine of the metal binding motif (Figure 3), which is pivotal for activity [16,18,22]. The exact chemical role of this arginine in catalysis has not yet been delineated. Its importance in hydrolysis most likely stems from the ability of this residue to ensure proper substrate positioning and/or to form intermolecular interactions with the substrate phosphate groups. Most biochemically characterized HD-domain mono- and diphosphatases are dimers in solution [3,5,16], which appears to be related to enzymatic function and may represent a regulatory mechanism for tuning activity. However, it is currently unknown if both sites are catalytically active or if one site allosterically activates the other. In contrast, triphosphatases are allosterically regulated by nucleotide binding to secondary sites, and most are active as tetramers [4,20] or hexamers [38].

In most cases, phosphate hydrolysis is supported by non-redox metal ions, with the most active cofactors being Co or Mn for monophosphatases, Mn for diphosphatases, and Mg or Mn for triphosphatases (Table 1). With a few notable exceptions (YqeK, SAMHD1, and OxsA), these metals are bound in a mononuclear configuration by a conserved “H … HD … D” motif (Figure 3).

#### 1.2.1. Monophosphatases

##### YfbR and YGK1

YfbR is a dimeric 5′-deoxyribonucleotidase that is specific to 2′-deoxyribonucleotide-5′-monophosphates (dNMPs) and hydrolyzes dAMP with a k_cat_/K_M_ of 25.6 × 10^3^ M^−1^s^−1^ [3]. YfbR is most active with a mononuclear Co^2+^ cofactor, while Mn^2+^ and Cu^2+^ can also support activity to a lesser extent [3]. The Co ion is bound in an octahedral geometry by the HD-domain residues H33, H68, D69, and D137, the phosphate oxygen of the nucleotide, and a water molecule that is activated by Co^2+^ for nucleophilic attack of the phosphate group. YfbR shows no preference for the chemical nature of the substrate base because the latter makes no significant interactions with the protein [3]. However, YfbR exhibits specificity for deoxyribomonophosphates, afforded by steric hindrance from W19 and additional hydrogen bond contacts between the deoxyribose moiety with residues R18 and D77. Substitutions of R18, D77, W19, and the metal binding ligands completely inactivate the enzyme [3].

The HD-domain monophosphatase YGK1 has high structural similarity to YfbR and acts on dNMPs with catalytic efficiencies between 0.78 × 10^3^ and 6.7 × 10^3^ M^−1^s^−1^ [5]. Similar to YfbR, the cofactor is mononuclear and held to the protein polypeptide by H61, H89, D90, and D159 (Figure 3). The metal ions that support catalysis follow the trend: Mn^2+^ > Co^2+^ > Mg^2+^ [5].

#### 1.2.2. Diphosphatases

##### YqeK

YqeK is an Ap_4_A hydrolase that symmetrically cleaves Ap_4_A to two adenosine diphosphates (ADPs) with a k_cat_/K_M_ of 34 × 10^6^ M^−1^s^−1^ [10]. Increased levels of Ap_4_A are linked to temperature sensitivity, stress response, and antibiotic resistance [43]. Other substrates include Ap_5_A, Gp_4_G, Ap_4_G, and Ap_4_U with k_cat_/K_M_ values of 1.5 ×10^6^, 15 × 10^6^, 11 × 10^6^, and 18×10^6^ M^−1^s^−1^, respectively [10]. These catalytic efficiencies are similar to those observed by the ApaH family of Ap_4_A hydrolases, which are thought to be mutually exclusive from YqeK (i.e., *apaH* and *yqeK* genes do not co-occur in the same organism) [10].

YqeK attains a homodimeric configuration, and unlike other known HD-domain phosphatases, it binds a dimetal cofactor that, on the basis of the crystal structure, is diiron (Figure 3). The ligands to the diiron site include the conserved H21, H50, D51, and D127 residues as well as two additional histidines (H83, H109) that help coordinate the second metal ion (Figure 3) [5]. Thus, the ability to coordinate a dinuclear site can be diagnosed on the basis of primary sequence by the presence of six conserved residues making up the extended “H … HD … H … H … D” motif. The relevance of the diiron site in catalysis has not yet been explored, and the chemical nature of the active cofactor is presently unknown.

##### YpgQ

YpgQ and its orthologs belong to a smaller subfamily within the HD-domain phosphatases, which is designated as RnaY (IPR017705), that contain the RNase Y N-terminal region. While several of its homologs have been crystallographically characterized, YpgQ is the sole representative that has been biochemically characterized [16]. It is a homodimer that hydrolyzes oxy and deoxy nucleotide triphosphates (NTPs and dNTPs) and preferentially acts on dGTP and dATP with apparent k_cat_/K_M_ values of 281 and 91 M^−1^s^−1^, respectively [16]. It harbors a catalytically essential mononuclear Mn cofactor coordinated by the invariant residues H29, H58, D59, and D124. Four other residues in the vicinity of the active site (R136, H158, F140, and H25) are proposed to contribute to substrate specificity [16]. R136 and H158 make hydrogen bond contacts with the 3′ and 2′ hydroxyl groups of the ribose sugar, respectively. Additionally, H158 and F140 stabilize the purine base via π–π stacking, while H25 hydrogen bonds to the phosphate group such that the β-phosphate is bound to a metal-activated water molecule (Figure 3).

##### SpoT/RelA

SpoT and RelA act on the secondary messengers (p)ppGpp, which are alarmones produced as part of the stringent response to amino acid starvation, temperature change, and other environmental cues (Figures 2 and 3) [17,44]. Both proteins are believed to be dimers in solution [9]. SpoT is an Mn^2+^-dependent bifunctional enzyme with weak (p)ppGpp synthetic activity from its SYNTH domain and strong hydrolytic activity from its HD-domain [42,45]. RelA has only synthetic activity and lacks hydrolytic activity, which is most likely due to a divergent HD-domain (compared to that of SpoT) [42].

#### 1.2.3. Triphosphatases

##### SAMHD1

The sterile alpha motif and HD-domain containing protein 1 (SAMHD1) is a sophisticated deoxynucleotide triphosphate triphosphohydrolase that regulates dNTP levels in mammalian cells and plays a key role in HIV restriction, innate immunity, cancer, and cell cycle [2,4,46]. It does so by depleting cellular dNTP levels via an allosterically driven mechanism [47]. It has a strict specificity for deoxyribonucleotides (dGTP > dCTP > dTTP > dATP) [18] but can also hydrolyze other substrates to a lesser extent, including Clofarabine-TP, Ara-CTP, Ara-ATP, and Ara-UTP [48]. Hydrolysis of dNTPs by SAMHD1 is Mg-dependent and occurs with an apparent catalytic constant of 1–2 s^−1^ [49], but the protein is also active with Mn. Optimal activity is contingent on the assembly of a homotetrameric complex that is afforded by the sequential binding of oxy and deoxyribonucleotides that act as allosteric activators [48]. SAMHD1 has two allosteric (AL) sites; AL-site 1 (D137, Q142, and R145) is tuned for binding both oxy and deoxy guanosine-based nucleotides, while AL-site 2 (N119, D330, N358, and R372) is selective for dNTPs [4].

The main catalytic core is held to the protein polypeptide by four invariant residues, H167, H206, D207, and D311, which until recently was well accepted to bind a single metal ion [18,19]. A new structure, however, shows that the active site might be binuclear harboring a Fe–Mg center with the H233 coordinating the second metal ion (i.e., Mg) (Figure 3). The two metal ions are bridged by D207 and the substrate α-phosphate [19]. Two histidine residues vicinal to the active site, H210 and H215, form salt bridges with the phosphate oxygens [18], and together with R164 and H233, are important for catalytic activity [18].

##### EF1143

EF1143 from *Enterococcus faecalis*, a bacterium found in the human intestinal tract of humans, regulates the cellular levels of DNA building molecules by depleting the dNTP pools [20]. It is a distant homolog of SAMHD1 and shares some structural and functional similarities to the latter. Both SAMHD1 and EF1143 are homotetramers, and their first allosteric site is specific to guanosine-based triphosphates [4,20,48]. However, there are several salient differences between the two enzymes. EF1143 does not require dNTP binding for tetramerization, and in contrast to SAMHD1, the first allosteric site of EF1143 is strictly specific for dGTP [4,20]. The active site is similar to that of SAMHD1, harboring residues H66, H110, D111, and D183 to coordinate a divalent metal ion. The Mg form of EF1143 is activated by dGTP and selectively hydrolyzes dATP and dCTP. In contrast, the Mn form does not require a nucleotide effector and hydrolyzes all dNTPs [20].

##### OxsA

OxsA catalyzes the formation of the antitumor, antiviral, and antibacterial nucleoside analogue oxetanocin-A (OXT) [22] (Figure 2). OxsA employs H31, H66, D67, and D132 to bind its metallocofactor for the sequential hydrolysis of phosphate groups from phosphorylated forms of OXT (i.e., OXT-P, OXT-PP, and OXT-PPP). The crystallographically observed cofactor nuclearity is dependent on the extent of substrate phosphorylation such that a mononuclear form catalyzes the hydrolysis of the singly phosphorylated OXT-P, while the binding of a second metal ion extends the hydrolytic capabilities to act on OXT-PP and OXT-PPP [22]. OxsA is most active with Co^2+^ with apparent k_cat_/K_M_ values of 106, 221, and 525 M^−1^s^−1^ for OXT-P, OXT-PP, and OXT-PPP, respectively [22].

When OXT-P is bound, H66 occupies multiple orientations and can be replaced by water as a ligand to the metal ion. In contrast, when OXT-PP(P) is bound, H66 is a ligand to the active site metal. The second metal ion is octahedrally coordinated by D132, which bridges the two metal ions, water molecules, and the β and γ phosphate groups (Figure 3) [22]. Thus, it is hypothesized that upon sequential hydrolysis of the γ and β phosphates, OxsA loses the second metal ion, allowing OXT-P to occupy the second metal site for efficient hydrolysis. The substrate is stabilized via additional interactions between H75 and the C3′ hydroxymethyl group of OXT, as well as S78 and the endocyclic ring of OXT [22]. W17, a residue conserved both in OxsA and YfbR, is proposed to exclude ribonucleotide binding [22].

### 1.3. HD-Domain Hydrolases: The PDE Subfamily

**Figure 4.**
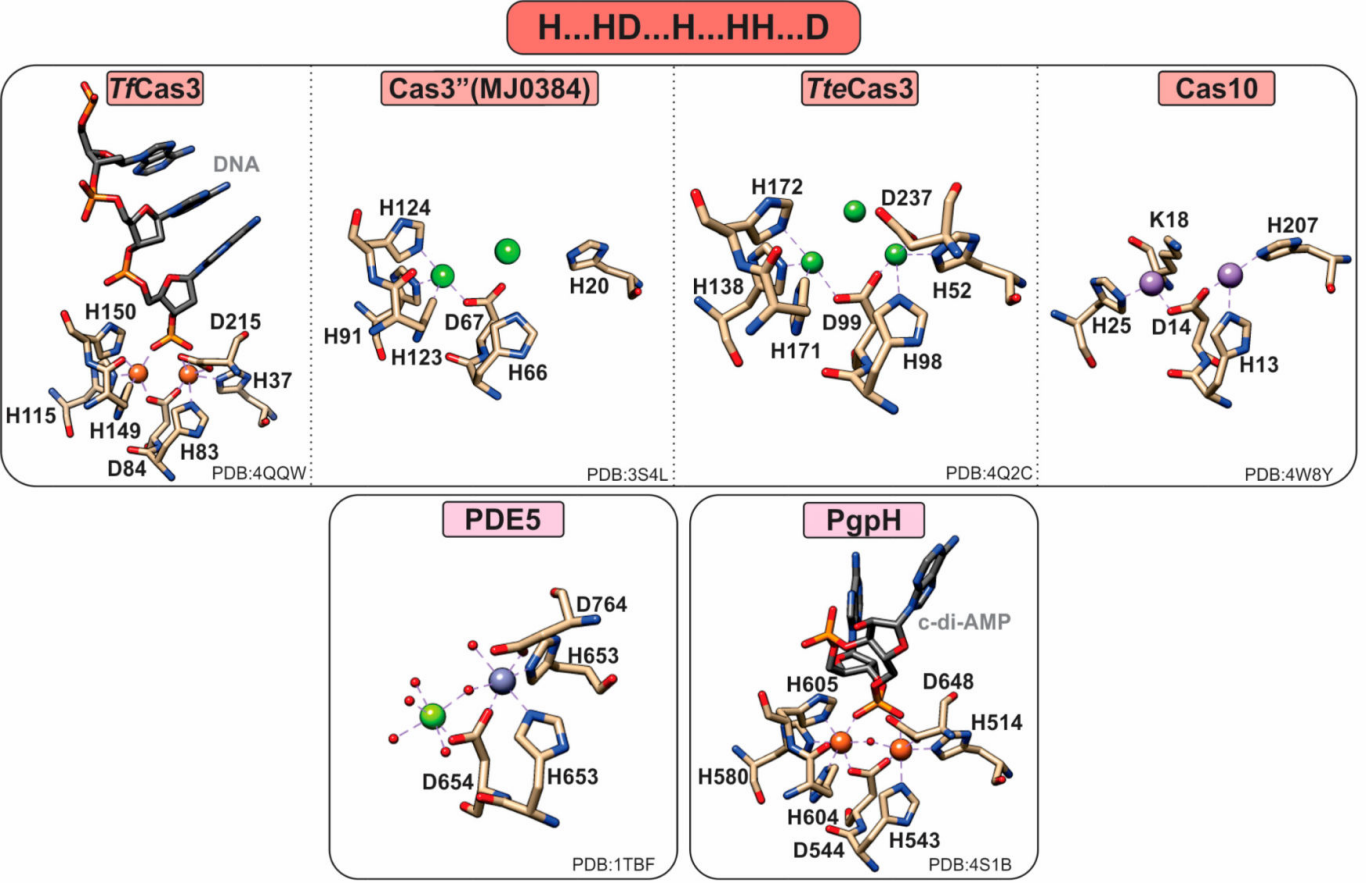
Active sites of HD-domain PDEs. HD-domain PDEs utilize the conserved HD motif “H…HD…H…HH…D” to bind a di- or trinuclear metal center. The metal ions coordinated in their active sites are zinc (purple), magnesium (light green), nickel (dark green), or iron (orange). Small red spheres represent water molecules.

PDEs can harbor both mononuclear and polynuclear cofactors. A common feature of all known polynuclear (di-or tri-nuclear) PDEs is an extra histidine residue in the active site such that their characteristic metal binding sequence is “H … HD … H … HH … D” (Figure 4). The role of this histidine in activity or structure has not been explicitly established. However, on the basis of structural studies, this residue makes additional hydrogen bond contacts to the substrate phosphate groups, suggesting a possible role in substrate binding.

#### 1.3.1. HD-Domain PDEs Acting on DNA

Clustered regularly interspaced short palindromic repeats (CRISPR)-associated systems (Cas) are major players in prokaryotic adaptive immunity and RNA-based defense [50,51]. Type I CRISPR–Cas utilize a multicomponent system and recruit a single nuclease, Cas3, for the degradation of invader nucleic acids. The Cas3-associated gene can encode for a protein that has only the HD-domain (Cas3′’, I-A subtype), or more commonly, an N-terminal HD-domain fused to a Superfamily 2 helicase (Cas3). Type III-B CRISPR–Cas utilize Cas10 (Cmr2) for RNA-activated ssDNA cleavage [26].

The crystal structure of the *Thermobifida fusca* Cas3 shows a diiron active site (Figure 4), yet no activity with this cofactor has been demonstrated [23]. Cas3 proteins can be promiscuously activated by various metal ions, with most being activated by Ni or Co and, to a lesser extent, by other divalent metal ions (with the exception of Mg and Ca) [23,25]. In contrast, the *Pyrococcus furiosus* Cmr2 (dinuclear) and the Cas3′’ proteins (mononuclear in the published structures) exhibit an Mg-dependent PDE activity [23,25]. The nuclearity of the Cas3′’s may be an artifact due to the larger flexibility of the protein polypeptide, making binding of the second metal ion more transient. Of note, no correlation between helicase and PDE activities has been demonstrated to date.

#### 1.3.2. HD-Domain PDEs Acting on Cyclic Mononucleotides

cAMP- and cGMP-specific PDEs (also referred to as PDEases) are essential regulators in cyclic nucleotide-dependent signal transduction in diverse physiological processes including immune response, neuronal activity, hypertension, and inflammatory response [30,31,52]. Currently, 21 genes encoding human PDEases have been identified and classified into 12 families according to their substrate specificities, pharmacological properties, and tissue localization [31,53]. These are further divided into three groups: cAMP-specific (PDE4, PDE7, PDE8, and PDE12), cGMP-specific (PDE5, PDE6, and PDE9) [31,41], and dual-specific (PDE1, PDE2, PDE3, PDE10, and PDE11) [31].

All PDEases are dimeric and have a conserved catalytic carboxy terminal domain as well as a variable regulatory amino terminal domain [30,54]. However, PDEases are also active as monomers; therefore, the functional significance of dimerization remains unknown [54]. On the basis of their binding affinity for divalent metal ions, PDEases are distinguished into two classes: Class I in mammals and flies and Class II in yeast and protozoans [55]. The most extensively studied are Class I PDEases, in which two metal ions (e.g., Zn and Mg) are octahedrally coordinated, forming a somewhat unconventional bimetallic site [30] Although the canonical motif suggests the binding of a single metal ion (M1) in the HD motif, stabilization of the second metal ion (M2) is accomplished via the aspartate of the HD motif and five water molecules, one of which is bridging M1 and M2. The bridging water molecule has been suggested to be a hydroxide, which can nucleophilically attack the phosphodiester bond [30]. In the crystal structures, the identity of M1 and M2 is often found to be Zn and Mg, respectively (Figure 4) [30]. Class I PDEs are active with either Mn or Mg but not with Zn. Therefore, although Zn can bind in the M1 position with high affinity, it cannot stimulate activity by itself or has inhibitory effects [31].

The substrate specificity in PDEases is afforded by a so-called “glutamine-switch” mechanism in which [30] a conserved glutamine in the vicinity of the active site can adopt two different orientations. In one orientation, it can form a bidentate hydrogen bond with the adenine ring (cAMP-specific) or two hydrogen bonds with guanine ring and two hydrogen bonds with neighboring alanine and tryptophan residues (cGMP-specific) [30,31]. In the dual-specific PDEases, the conserved glutamine has higher rotational flexibility and no orientation constraints, allowing it to adopt orientations for both substrates.

#### 1.3.3. HD-Domain PDEs Acting on c-di-AMP

Cyclic-di-AMP is a second messenger essential in bacterial signaling and a critical player in bacterial physiology and pathogenesis [56,57]. PgpH performs the one-step hydrolysis of c-di-AMP to 5′pApA in an Mn-dependent fashion but cannot hydrolyze other cyclic dinucleotides (i.e., c-di-GMP) [6]. The active site Mn^2+^ ions are octahedrally coordinated by seven residues, H514, H543, D544, H580, H604, H605, and D648, as well as the two terminal oxygen atoms of the c-di-AMP phosphate group (Figure 4). The metal ions activate a water molecule opposite the scissile phosphate for the nucleophilic attack of phosphorus. Protonation of the resulting oxyanion terminates the reaction [6].

#### 1.3.4. HD-Domain PDEs Acting on c-di-GMP and c-GAMP; the HD-GYP Subclass

HD-GYPs are a special subclass of the PDE subfamily and are functionally homologous to EAL proteins (typified by the glutamate-alanine-leucine residue triad) [1,58]. They can be single domain proteins or fusions to extra regulatory, sensory, or catalytic protein domains [8,59,60].

While cyclic-di-GMP is their most common substrate, the recently discovered hybrid dinucleotide, 3′3′c-GAMP, is also hydrolyzed by some HD-GYPs [59]. Out of the nine HD-GYPs encoded in *Vibrio cholerae*, VCA0681, VCA0931, and VCA0210 are the only HD-GYPs to hydrolyze both c-di-GMP and 3′3′c-GAMP. More recently, PmxA from *Myxococcus xanthus* was identified as a 3′3′c-GAMP specific PDE that is hardly active toward c-di-GMP or c-di-AMP [60]. Selectivity for 3′3′c-GAMP is attributed to a glutamine near the active site, although this residue is not conserved in VCA0681, VCA0931, and VCA0210, suggesting that the molecular origins for 3′3′cGAMP specificity may vary among HD-GYPs.

**Figure 5.**
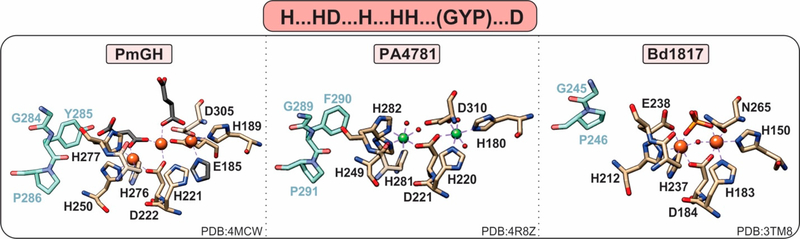
Active site of HD-GYPs. HD-GYP proteins utilize an “H…HD…H…HH…D” motif that typically binds a dinuclear metal center. The third metal ion in the PmGH active site is stabilized by crystallization molecules shown in gray. In addition, these enzymes contain a GYP residue triad vicinal to the active site (shown in blue), the importance of which is currently unclear. Bd1817, which is inactive toward c-di-GMP, lacks the GYP tyrosine and the terminal aspartate is an asparagine.

In addition to the seventh ligand added to their active site (i.e., an extra histidine adjacent to the last histidine of the motif), all active HD-GYPs have a glycine-tyrosine-proline (GYP) residue triad in a loop close to the active site (Figure 5) [14,35]. However, because single amino acid substitutions of each of the GYP domain residues to alanines hardly affect PDE activity [36], its role in catalysis and protein stability remains poorly understood. The GYP motif is considered important for interaction with the GGDEF cyclase (named after its highly conserved Gly-Gly-Asp-Glu-Phe sequence motif) [8] and serves as a substrate specificity element for the recognition of c-di-GMP and its hybrid 3′3′-cGAMP analog [40].

HD-GYPs differ on the basis of their active metal cofactor and possible catalytic outcomes. While most commonly harbor a dimetal cofactor, some incorporate a trinuclear cofactor by involving a glutamate residue as an eighth ligand to the site (Figure 5) [36]. The assembly of a dinuclear or trinuclear cofactor is presumed to afford different reaction outcomes. Dinuclear HD-GYPs can only perform a one-step hydrolysis, whereas trinuclear ones can perform a two-step hydrolysis, leading to the respective monophosphates. Metal ions that can stimulate hydrolysis are Fe, Mn, and, to a lesser extent, Co and Ni [40].

PmGH from *Persephonella marina* is the prototypical trinuclear HD-GYP and the first to be crystallographically characterized [36]. PmGH harbors a triiron cofactor with the third iron coordinated by the glutamate E185. The trimetal cofactor is additionally stabilized by three other crystallization molecules, invoking the possibility that other solvent molecules may be incorporated under physiological conditions (Figure 5). It is active with both Fe^2+^ and Mn^2+^. On the basis of primary amino acid sequence, PA4781 from *Pseudomonas aeruginosa* is also a putative trinuclear PDE; however, the available crystal structure shows two Ni ions in the active site at an elongated distance. PA4781 is unselective in its metal ion incorporation, has limited activity, and exhibits a preference for 5′-pGpG over c-di-GMP to form GMPs [14].

Only one structure of a dinuclear HD-GYP exists: Bd1817 from *Bdellovibrio bacteriovorus*. It harbors a diiron cofactor, but the presence of an asparagine instead of the last aspartate of the binding motif, a degraded GYP motif (Figure 5), and its complete inactivity toward c-di-GMP [35] do not allow for the inference of substrate positioning and specificity in dinuclear HD-GYPs.

### 1.4. HD-Domain Oxygenases

Most of the known HD-domain proteins are phosphohydrolases, but three members, namely *myo*-inositol oxygenase (MIOX), PhnZ, and TmpB, are monooxygenases and perform the oxidative cleavage of a C-X bond [11–13,15]. The discovery of this chemistry expands the catalytic repertoire of the HD-domain superfamily, and their conserved protein features may provide insight into the identification of yet-to-be-characterized HD-domain proteins as oxygenases.

**Figure 6.**
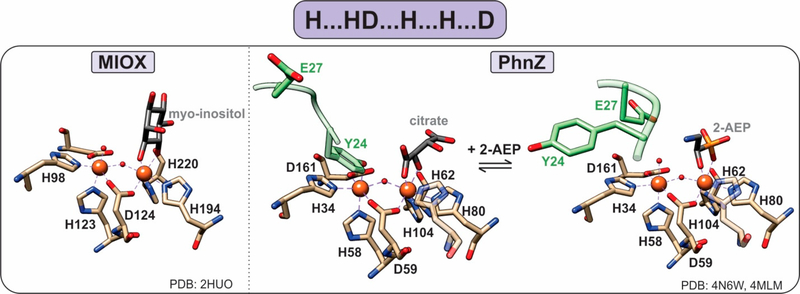
Active sites of HD-domain oxygenases. Oxygenases utilize the “H … HD … H … H … D” motif to bind a diiron metal center. The substrate scissile bond is positioned above one of the iron sites, leaving the second site open for oxygen binding. PhnZ and TmpB contain an YxxE loop (green) in their primary sequence that is located vicinal to the active site, which upon substrate binding undergoes a conformational change to allow for oxygen binding and catalysis.

The first discovered HD-domain oxygenase, MIOX, catalyzes the oxidative cleavage of a C-C bond of *myo*-inositol to form D-glucuronic acid (Figure 6) [11]. *Myo*-inositol is a precursor for inositol phosphoglycans, which act as insulin mediators, and altered inositol metabolism has been associated with diabetes. Therefore, the activity of MIOX is of increasing interest, as it presents a potential therapeutic target for treating both type-1 and type-2 diabetes.

PhnZ and TmpB were later established as oxygenases, demonstrating that MIOX is not a functional outlier [12,13]. Both PhnZ and TmpB are involved in the degradation of organophosphonates, which are compounds that serve as sources of inorganic phosphate for bacteria that occupy phosphate-limited environments (e.g., marine ecosystems) [12]. Unlike MIOX, PhnZ and TmpB act in tandem with the non-heme α-ketoglutarate (KG) dependent hydroxylases PhnY and TmpA, respectively, to cleave the C–P bond of their substrates (Figure 2) [12,13]. PhnY initiates the degradation of 2-aminoethylphosphonate (2-AEP) via the addition of a hydroxyl group to the C1 carbon in a stereospecific manner producing (*R*)-2-amino-1-hydroxyethyl phosphonate (OH-AEP) [12]. PhnZ performs the subsequent oxidative cleavage of the C-P bond of OH-AEP forming inorganic phosphate and glycine (Figures 2 and 6) [12]. The TmpA/TmpB pathway is mechanistically similar, with the only difference being the nature of the substrate, i.e., 2-(trimethylammonio)ethyl phosphonate (TMAEP) for TmpA [13].

MIOX, PhnZ, and TmpB bind a catalytically essential diiron cofactor via the HD-domain sequence “H … HD … H … H … D” (Figure 6). Each iron is coordinated in an octahedral geometry and bridged by the carboxylate group of the first aspartate residue in the HD-domain sequence as well as a μ-oxo/hydroxo bridge (Figure 6) [15]. Unlike other dinuclear nonheme–iron oxygenases, which utilize the fully reduced Fe^II^/Fe^II^ form of their cofactors, HD-domain oxygenases stabilize a mixed-valent Fe^II^/Fe^III^ state for the four-electron oxidation of a C-C/P bond and do not require an external reducing system for reactivation of the cofactor (i.e., after one substrate turnover the site returns to the Fe^II^-Fe^III^ form) [15]. Stabilization under the same redox conditions of the Fe^II^Fe^III^ cofactor in oxygenases and the Fe^II^Fe^II^ cofactor in HD-domain hydrolases suggests that the HD-domain sites may tune activity through the modulation of cofactor reduction potentials [40].

The iron ion in the Fe1 site serves as a Lewis acid and binds the substrate such that the C-X bond is opposite the iron (Figure 6) [15]. Then, the second iron site (Fe2) is available to bind molecular oxygen, forming a Fe^III^/Fe^III^ superoxo species that initiates oxidative cleavage by abstracting a hydrogen atom from the substrate. Following turnover, the active mixed-valent Fe^II^/Fe^III^ form is regenerated [12], and thus, there is no need for an external reducing system to reactivate the enzyme, which is a feature unique to HD-domain containing oxygenases.

Unlike MIOX, PhnZ and TmpB sequences contain a transient YxxE loop involved in substrate binding. Prior to substrate binding, the tyrosine (Y24 in PhnZ and Y30 in TmpB) is oriented toward the active site and binds to the Fe2 site, while the glutamate (E27 in PhnZ and E33 in TmpB) faces away from the active site [13,15]. Substrate binding induces a conformational change, positioning the glutamate within hydrogen bonding distance to the amino group of the substrate and causing the tyrosine–iron bond to break [13,15]. Dissociation of the tyrosine frees the Fe2 site for O_2_ binding and subsequent turnover and most likely serves as a protective mechanism to prevent oxidative inactivation of the active site (Figure 6).

Collectively, HD-domain oxygenases have catalytic and structural features that differ significantly not only from that of other nonheme–iron oxygenases, but also of HD-domain hydrolases. This divergence is useful as it can provide some descriptors to distinguish oxygenases from hydrolases within the HD-domain family. It is likely that these characteristics are conserved among all HD-domain oxygenases and may provide a critical first step into the characterization of other HD-domain proteins of unknown function.

### 1.5. Lyases

#### The HD-Domain Cyanamide Hydratase Ddi2

**Figure 7.**
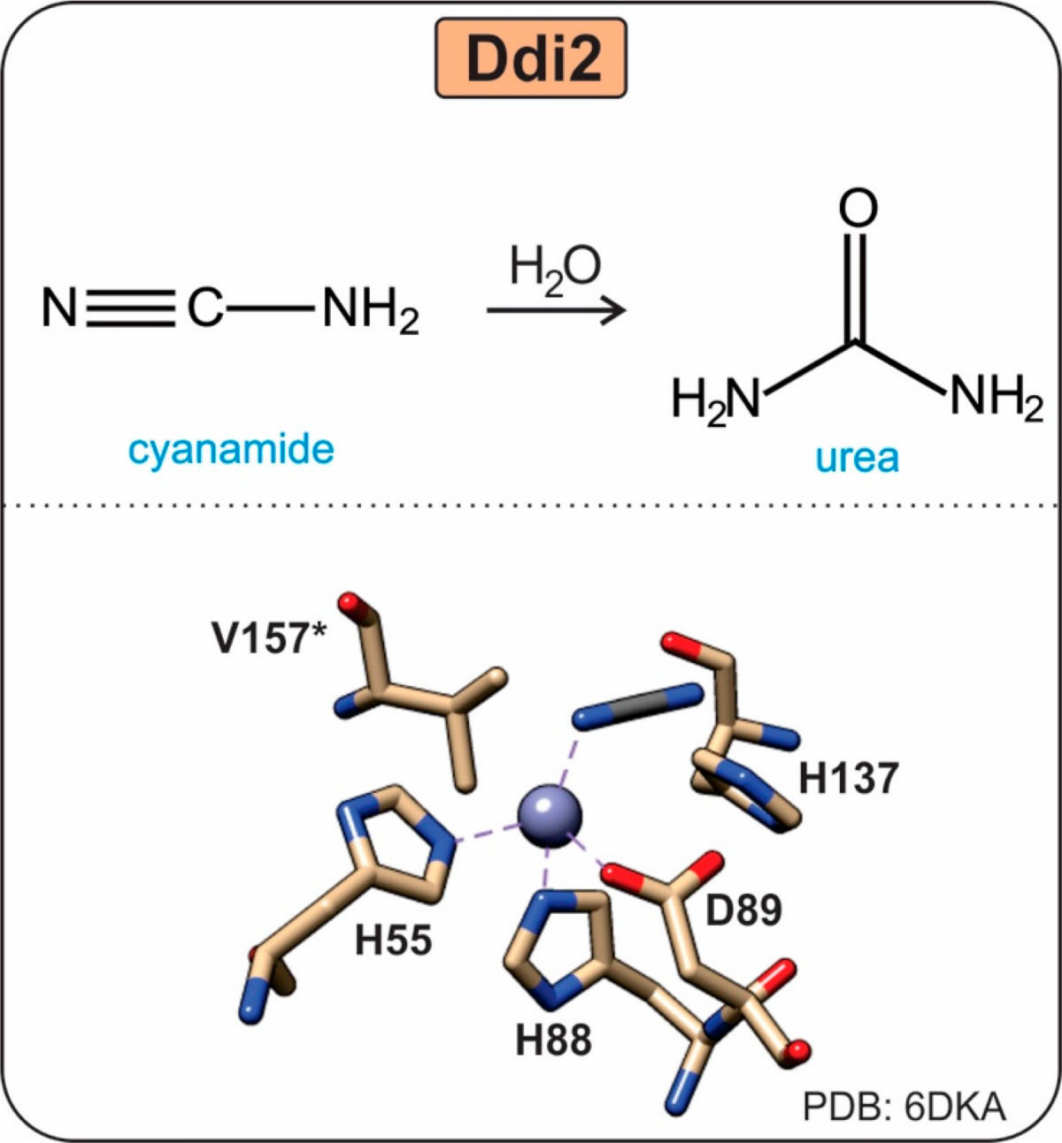
Reaction and active site of the HD-domain hydratase Ddi2. Ddi2 utilizes a Zn metal center to convert cyanamide to urea. The terminal aspartate residue found in HD-domain proteins is a replaced by a threonine in Ddi2. The role of this threonine, T157 (valine in the crystal structure), is predicted to allow for substrate positioning or metal specificity [37].

Cyanamide is a toxic nitrile compound used in agriculture both as a fertilizer and herbicide, as well as an alcohol deterrent for humans. Some soil-inhabiting microorganisms, such as *Myrothecium verrucaria* and *Saccharomyces (S.) cerevisiae*, have developed cyanamide biodegradation pathways as a likely defense mechanism against cyanamide-producing plants. The cyanamide hydratase Ddi2, although currently not well characterized, catalyzes the conversion of cyanamide to urea (Figure 7) and represents a functionally distinct subgroup of the HD-domain superfamily [37].

The *S. cerevisiae* Ddi2 has a unique HD metal binding motif, “H … HD”, in which the characteristic second aspartate residue is absent and T157 occupies that position. Ddi2 hydrates cyanamide with a catalytic efficiency of 566 M^−1^s^−1^ and utilizes residues H55, H88, and D89 to coordinate the active site Zn^2+^ [37]. The binding of cyanamide to the active site displaces a water molecule for direct coordination to the Zn^2+^ via a substrate nitrogen atom. It remains unclear whether substrate binding occurs by the amino nitrogen or the nitrile atom. However, it is likely that the nitrile atom binds the Zn directly in a similar fashion to carbonic anhydrase [37].

## 2. Conclusions

The HD-domain superfamily is multi-faceted with respect to its functional repertoire and structural diversity. Although apparently similar, HD-domain proteins can catalyze a variety of different reactions by incorporating protein-based and inorganic-based elements that tune their activities. The present review demonstrates that, to date, identification of the cofactor nuclearity, chemical nature, and substrate selectivity cannot be inferred solely on the basis of primary amino acid sequence or crystallography. This fact may be ascribed both to the plasticity of the HD motif to accommodate non-canonical residues or solvent ligands as well as the promiscuity of the motif for the coordination of divalent (and trivalent) metal ions.

To date, there is no established correlation between the chemical nature of the metallocofactor and the type of the reaction performed by the HD-domain enzymes. The employment of an Fe-based cofactor was initially thought to occur solely in oxygenases and be unfavorable for hydrolases due to the redox-active nature of Fe. However, more and more hydrolytic enzymes are found to incorporate catalytically relevant Fe-based cofactors, revoking this notion. Whereas some HD-domain hydrolases are selective for the types of divalent metal ions that can stimulate activity (e.g., PDEs1–11 HD-GYPs), others are rather promiscuous and can utilize most of the first-row transition metal ions (e.g., Cas3 PDEs). It is still puzzling why the metal ion selectivity is wide and why some of the proteins are highly selective, while others are highly unselective, especially when considering the high degree of conservation of the HD-domain binding motif. It could be that metal ion selection is linked to the environmental niches of the organisms in which the related HD-domain proteins are found or could reflect the apparent independence of a specific reaction on the exact chemical nature of the metal ion. Overall, there appears to be a loose relationship between the type of cofactor and the type of reaction catalyzed. All currently reported HD-GYP PDEs are dinuclear (or trinuclear), highlighting perhaps the importance of both metal ions in substrate binding and hydrolysis. All presently known oxygenases are also dinuclear. On the basis of their structure and mechanism, one of the metal ions is employed in molecular oxygen binding and the second one is involved in cosubstrate binding. However, cofactor selection on the basis of activity type breaks down for phosphatases, as both mono- and dinuclear active site configurations can act on similar (if not the same) substrates. There is an observed trend, in which bulkier substrates or compounds with multiple hydrolyzable bonds are most likely to be processed by multinuclear HD-domain enzymes. Presumably, in these cases the extended active site structure facilitates substrate binding and catalysis.

The diversity of the HD-domain metalloproteins is continuing to expand as more members become biochemically characterized, allowing for the identification of specific traits that can act as molecular descriptors and predictors of function. Within this ever-growing family, these features may aid in the assignment of HD-domain proteins with unknown activities as well as new hydrolases, oxygenases, lyases, or proteins of new unidentified chemistries.

## Figures and Tables

**Table 1.** List of representative histidine–aspartate (HD)-domain proteins from the three known subclasses, oxygenases, phosphatases and phosphodiesterases (PDEs), that are crystallographically and biochemically characterized.

Subclasses	Protein	Nuclearity	Active Metal	Chemistry	Substrate	PDB ID	Origin	References

**oxygenases**	MIOX	dinuclear	Fe	Oxygenase	myo-inositol	2HUO	*Mus musculus*	[11]
	PhnZ	dinuclear	Fe	Oxygenase	OH-AEP	4MLM	bacterium HF130_AEPn_1	[12,15]
	TmpB	dinuclear	Fe	Oxygenase	OH-TMAEP	6NPA	*Leisingera caerulea*	[13]

**phosphatases**	YfbR	mononuclear	Co	Monophosphatase	dAMP	2PAQ	*Escherichia coli* K-12	[3]
	YGK1	mononuclear	Mn	Monophosphatase	dNMP	5YOX	*Saccharomyces cerevisiae*	[5]
	YqeK	dinuclear	Fe	Diphosphatase	Ap_4_A	2O08	*Bacillus halodurans*	[10]
	YpgQ	mononuclear	Mn	Diphosphatase	dNTP	5DQV	*Bacillus subtilis*	[16]
	SpoT	mononuclear	Mn	Diphosphatase	(p)ppGpp	1VJ7	*Streptococcus dysgalactiae*	[17]
	SAMHD1	mononuclear	Mg	Triphosphatase	dNTP	3U1N	*Homo sapiens*	[18,19]
	EF1143	mononuclear	Mg	Triphosphatase	dNTP	4LRL	*Enterococcus faecalis* V583	[20,21]
	OxsA	Mono/dinuclear *	Co	Mono/Di/Triphosphatase	Oxetanocin-A	5TK8	*Bacillus megaterium*	[22]

**phosphodiesterases**	Cas3	dinuclear	Co	PDE	ssDNA	4QQW	*Thermobifida fusca* YX	[23]
	Cas3	dinuclear	Ni	PDE	ssDNA	4Q2C	*Thermobaculum terrenum*	[24]
	Cas3″	dinuclear	Ca	PDE	ssDNA	3S4L	*Methanocaldococcus jannaschii*	[25]
	Cas10	dinuclear	Ni, Mn	PDE	ssDNA	4W8Y	*Pyrococcus furiosus*	[26]
	PDE1-3	dinuclear ^#^	Mg, Mn	PDE	cAMP, cGMP	1TAZ, 3ITU, 1SO2	*Homo sapiens*	[27,28]
	PDE4	dinuclear ^#^	Mg, Mn	PDE	cAMP	1F0J	*Homo sapiens*	[29]
	PDE5	dinuclear ^#^	Mg	PDE	cGMP	1TBF	*Homo sapiens*	[30]
	PDE7-8	dinuclear ^#^	Mn	PDE	cAMP	4PM0, 3ECM	*Homo sapiens*	[31,32]
	PDE9	dinuclear ^#^	Mn, Mg	PDE	cGMP	3DY8	*Homo sapiens*	[33]
	PDE10	dinuclear ^#^	Mg	PDE	cAMP, cGMP	2OUN	*Homo sapiens*	[34]
	PgpH	dinuclear	Mn	PDE	c-di-AMP	4S1B	*Listeria monocytogenes*	[6]
	Bd1817	dinuclear	Fe *	PDE	c-di-GMP	3TM8	*Bdellovibrio bacteriovorus*	[35]
	PmGH	trinuclear	Fe, Mn	PDE	c-di-GMP	4MCW	*Persephonella marina* EX-H1	[36]
	PA4781	trinuclear ^&^	Mg	PDE	c-di-GMP	4R8Z	*Pseudomonas aeruginosa* PAO1	[14]

**lyases**	Ddi2	mononuclear	Zn	Hydratase	Cyanamide	6DKA	*Saccharomyces cerevisiae*	[37]

# Denotes proteins for which the crystal structure shows two active site metal ions at an average interatomic distance of ≈3.8 Å. The primary sequence suggests a mononuclear binding site. In phosphodiesterases (PDEs), the second metal ion is stabilized by the aspartate of the HD motif, a bridging hydroxide and four terminally ligated water molecules.

& Although no experimental evidence currently exists, PA4781 belongs the trinuclear clade of HD-GYP proteins as inferred from its primary amino acid sequence.

* Bd1817 is inactive toward Bis-(3′-5′)-cyclic guanosine monophosphate (c-di-GMP); therefore, the active metal ion refers to the metal ion observed in the crystal structure.

OH-AEP stands for 1-hydroxy-2-aminoethylphosphonate, OH-TMAEP stands for 1-hydroxy-2-(trimethylammonio)ethylphosphonate, dNMP stands for deoxymonophosphate, in which N can be A, G, U, C, Ap_4_A stands for diadenosine tetraphosphate, cGMP stands for guanosine 3′,5′-cyclic monophosphate, cAMP stands for adenosine 3′,5′-cyclic monophosphate, c-di-AMP stands for Bis-(3′-5′)-cydic adenosine monophosphate.
